# Time for a Standardized Common Femoral Artery Classification System

**DOI:** 10.1007/s00270-021-03034-6

**Published:** 2022-02-15

**Authors:** Thomas Zeller

**Affiliations:** Clinic for Cardiology and Angiology II, University Heart Center Freiburg – Bad Krozingen, Campus Bad Krozingen, Südring 15, 79189 Bad Krozingen, Germany

During the last decade, interventional therapy of common femoral artery (CFA) lesions has become an attractive alternative to the open surgical reconstruction by means of endarterectomy with or without patch plastic. However, prospective comparative data between endovascular treatment and surgery are still rare mainly comparing stenting with TEA [1]. CFA lesions may differ regarding location (exclusive CFA involvement vs. extension into the femoral bifurcation or external iliac artery) and morphology (e.g., degree of calcification). Therefore, there is a need for a standardized classification system to allow the comparison of published data as well as to identify treatment strategies that fit best individual lesion characteristics.

The modified coronary Medina classification proposed by Bonvini et al. is limited by considering only the anatomical lesion location and lesion extension [2]. The Azema classification included information about lesion morphology defining four types of lesion locations [3] but the degree of calcification and the severity of the lesion (stenosis vs. occlusion) were not part of the classification system. However, these missing parameters are potential predictors for acute treatment success and durability of the revascularization procedure.

In the present study, Rabellino et al. modified the Azema classification in a very practical way [4]: Previous Type IV lesions defined as bypass stenotic lesions were excluded in order to limit the new classification to native artery atherosclerotic lesions. Hence, Type I-III lesions remained the same. Type IV lesions in the new classification include lesions extending either from the external iliac artery (EIA) or common iliac artery (CIA) into the CFA and affecting its bifurcation.

Three additional lesion sub-characteristics were added for a more detailed lesion description of the four types of lesions: In Type III and IV lesions, it was specified which branch of the femoral bifurcation is involved. If only the superficial femoral artery (SFA) is affected, this was classified with an “S.” If only the deep femoral artery (DFA) was involved, a “D” was given, and if both branches were involved, it was classified as a “B” for both. As such, a Type III lesion starting at the CFA and extending to both the branches was denominated as a “TYPE III B” lesion. A further differentiation involves stenotic (“S”) versus occlusive (“O”) lesions. Finally, vascular calcium assessment was made by fluoroscopy or by computed tomography and added to the classification in heavy calcium burden “H” or with mild to moderate calcium burden “M.” Back to the example of a Type III B lesion with occlusive disease and heavy calcium burden, the lesion would be classified as a Type III B, O, H lesion.

Classifying CFA lesions in this proposed fashion in upcoming studies will allow a better comparison of acute and potentially long-term outcomes. Besides the small sample size, the main limitation of the study is the unusual definition of primary patency (“time free from more than 50% restenosis following index treatment”). This makes it impossible to compare the primary study end point of the Rabellino with historic studies and limits the prediction of long-term treatment success stratified to the proposed lesion characteristics.

In summary, the new CFA lesion classification proposed by Rabellino et al. should be applied to all upcoming studies for improving study comparability and identification of potential predictors of acute and long-term treatment success for each individual treatment modality.
